# Decision Support for Managing Common Musculoskeletal Pain Disorders: Development of a Case-Based Reasoning Application

**DOI:** 10.2196/44805

**Published:** 2024-05-10

**Authors:** Fredrik Granviken, Ottar Vasseljen, Kerstin Bach, Amar Jaiswal, Ingebrigt Meisingset

**Affiliations:** 1 Department of Public Health and Nursing Norwegian University of Science and Technology Trondheim Norway; 2 Clinic of Rehabilitation St Olavs Hospital Trondheim Norway; 3 Department of Computer Science Norwegian University of Science and Technology Trondheim Norway; 4 Unit for Physiotherapy Services Trondheim Municipality Trondheim Norway

**Keywords:** case-based reasoning, musculoskeletal pain, physiotherapy, decision support, primary care, artificial intelligence

## Abstract

**Background:**

Common interventions for musculoskeletal pain disorders either lack evidence to support their use or have small to modest or short-term effects. Given the heterogeneity of patients with musculoskeletal pain disorders, treatment guidelines and systematic reviews have limited transferability to clinical practice. A problem-solving method in artificial intelligence, case-based reasoning (CBR), where new problems are solved based on experiences from past similar problems, might offer guidance in such situations.

**Objective:**

This study aims to use CBR to build a decision support system for patients with musculoskeletal pain disorders seeking physiotherapy care. This study describes the development of the CBR system SupportPrim PT and demonstrates its ability to identify similar patients.

**Methods:**

Data from physiotherapy patients in primary care in Norway were collected to build a case base for SupportPrim PT. We used the local-global principle in CBR to identify similar patients. The global similarity measures are attributes used to identify similar patients and consisted of prognostic attributes. They were weighted in terms of prognostic importance and choice of treatment, where the weighting represents the relevance of the different attributes. For the local similarity measures, the degree of similarity within each attribute was based on minimal clinically important differences and expert knowledge. The SupportPrim PT’s ability to identify similar patients was assessed by comparing the similarity scores of all patients in the case base with the scores on an established screening tool (the short form Örebro Musculoskeletal Pain Screening Questionnaire [ÖMSPQ]) and an outcome measure (the Musculoskeletal Health Questionnaire [MSK-HQ]) used in musculoskeletal pain. We also assessed the same in a more extensive case base.

**Results:**

The original case base contained 105 patients with musculoskeletal pain (mean age 46, SD 15 years; 77/105, 73.3% women). The SupportPrim PT consisted of 29 weighted attributes with local similarities. When comparing the similarity scores for all patients in the case base, one at a time, with the ÖMSPQ and MSK-HQ, the most similar patients had a mean absolute difference from the query patient of 9.3 (95% CI 8.0-10.6) points on the ÖMSPQ and a mean absolute difference of 5.6 (95% CI 4.6-6.6) points on the MSK-HQ. For both ÖMSPQ and MSK-HQ, the absolute score difference increased as the rank of most similar patients decreased. Patients retrieved from a more extensive case base (N=486) had a higher mean similarity score and were slightly more similar to the query patients in ÖMSPQ and MSK-HQ compared with the original smaller case base.

**Conclusions:**

This study describes the development of a CBR system, SupportPrim PT, for musculoskeletal pain in primary care. The SupportPrim PT identified similar patients according to an established screening tool and an outcome measure for patients with musculoskeletal pain.

## Introduction

### Background

Musculoskeletal pain conditions are the leading cause of disability and a major societal burden worldwide [1]. Common interventions for musculoskeletal pain either lack evidence to support their use or, at best, have modest or only short-term effects [2,3]. Treatment guidelines are based on randomized controlled trials considering effects on the group level with little or no consideration for the huge variation in patient stories and individual symptoms, even within more narrowly defined diagnostic entities, for example, low back pain. Thus, applying group-level evidence to individual patients and the relevance of one-size-fits-all treatment guidelines have been questioned [4]. In addition, the highly selected patients in most clinical trials do not match clinical settings where patients often present with comorbidities and large variations in symptoms and clinical history. Thus, clinicians are at unease with and often do not follow evidence-based guidelines [5]. Different attempts at subgrouping patients have been explored [6,7], but most attempts of subgrouping patients according to symptoms and clinical characteristics, and offering matched treatments (stratified care), have yet to demonstrate superior treatment outcomes [8].

It has been argued to focus less on diagnostic classification in musculoskeletal pain and more on prognostic factors to inform treatment decisions and improve treatment outcomes [9]. Factors influencing patients’ course and treatment outcomes are many, making decisions on the best treatment approach challenging for clinicians. In this situation, artificial intelligence (AI) may add decision support [10]. An intriguing AI method relevant to musculoskeletal pain disorders is case-based reasoning (CBR), where experiences from past problems and their solutions are used to solve new problems [11]. CBR may advance decision-making for musculoskeletal pain disorders and improve patient care and outcomes by providing information for tailoring the treatment. CBR has been used in fields such as the oil industry [12]; weather prediction [13]; and different aspects of health care [14], for example, kidney functioning in an intensive unit setting [15], assessment and diagnosis of depression in palliative care [16], follow-up of patients who underwent stem cell transplantation [17], and diabetes management [18]. More recent studies have used CBR in diagnostics [19] and promoting self-management [20,21]. Yin et al [19] developed a CBR-based decision support system capable of differentiating between 2 types of probable primary headaches challenging for physicians in clinical care. The system was found to have high accuracy and differentiated probable migraine and probable tension-type headache much better than a guideline-based system. In a previous study from our research group, CBR was used to capture patient experiences and find the best treatment advice for patients by evaluating how to carry out a similarity-based retrieval [22]. This was further used for tailoring self-management support for patients with low back pain through a smartphone app. The app was provided as an adjunct to usual care and compared to usual care only in a randomized controlled trial. The patients in the intervention group reported a larger improvement in disability compared to those receiving usual care only [20]. The effect of the app has recently also been tested in a 3-armed randomized controlled trial among patients with neck and back pain in the specialist care. The authors reported no differences in effects among use of the app, a web-based nontailored self-management support tool, or usual care alone [21].

### Objective

In this study, we used CBR to build a system for decision support in patients with musculoskeletal pain seeking physiotherapy care. This study describes the development of the system and demonstrates the system’s ability to identify similar patients.

## Methods

### The CBR Cycle Versus Physiotherapy Way of Solving a Problem

**Figure 1:**
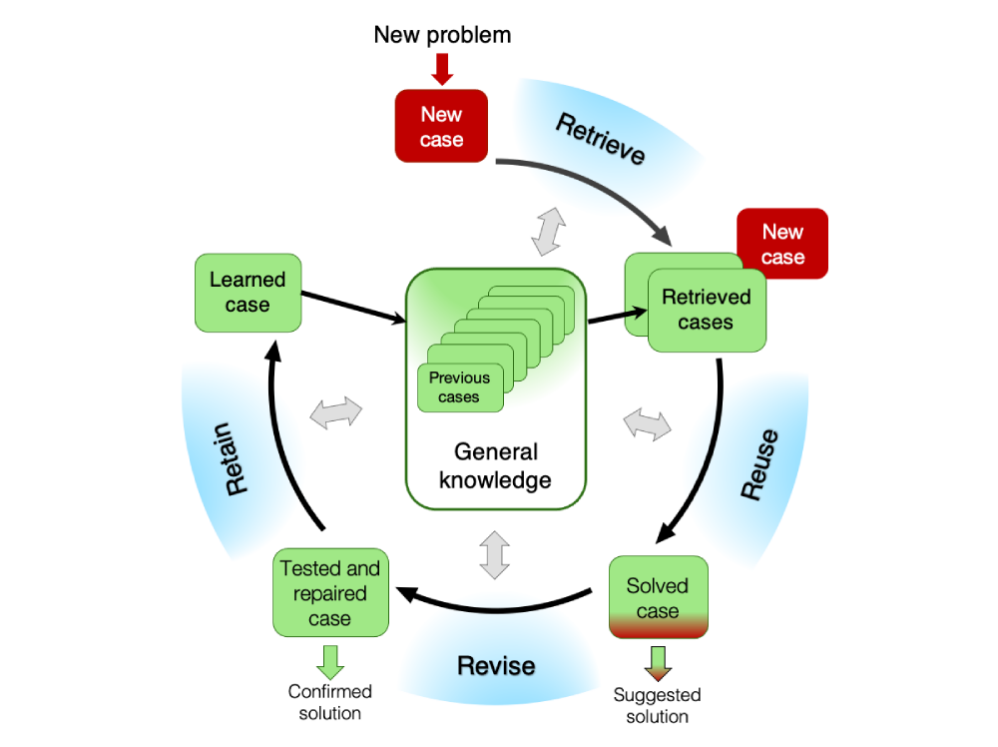
The case-based reasoning cycle, adapted from the study by Aamodt and Plaza [11].

CBR has been described as a 4-step process, known as the CBR cycle: retrieve, reuse, revise, and retain (Figure 1) [11]. The most similar case or cases are *retrieved* from the collection of previous cases (stored in the case base), where a case is a set of data that represents a problem with its solution from the past. Knowledge of the CBR model (eg, adaptation rules) is applied to fit a new problem to an existing solution (reuse). The solution for the new case is tested for success and *revised* if necessary. The system learns as useful experiences of the new case are *retained* for future problem-solving, such that the case base is continuously updated with new or modified cases. Building and refining the collection of cases, the case base, is an important step in the CBR process [23]. The CBR methodology assumes that similar problems have similar solutions. Translated to medical terms, the *problem* is defined by a detailed description of the patient’s characteristics, signs, and symptoms, and the *solution* is defined by the treatment leading to a successful outcome. New patients are matched to previous similar patients (*problems*) with a successful outcome, and their treatment is used to inform treatment for the new patient (*solution*).

**Figure 2:**
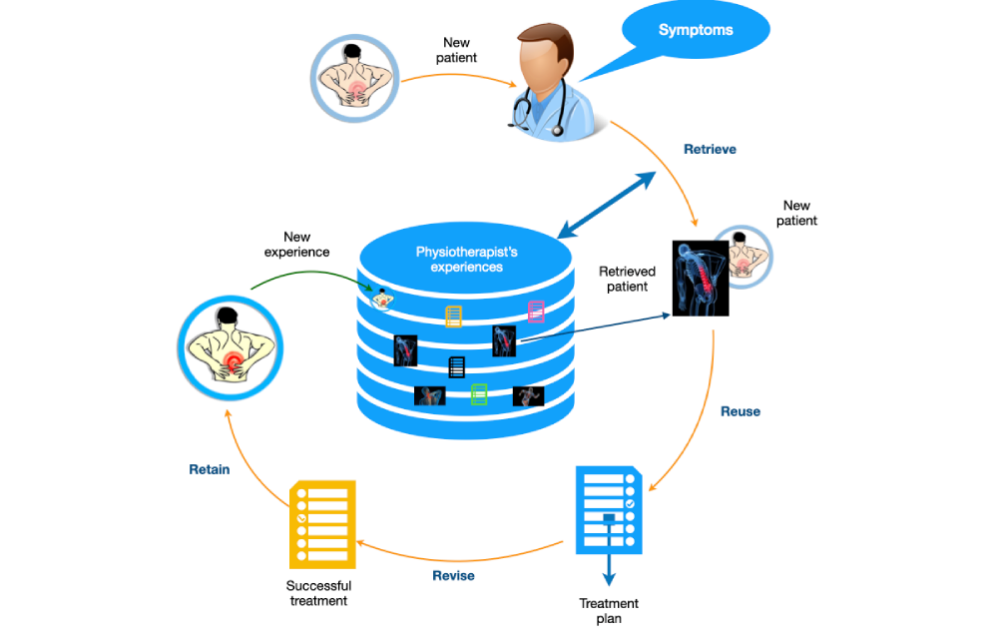
Physiotherapist’s way of solving a problem (ie, how to treat a new patient).

An important reason for choosing CBR as the AI method of choice in this study (Figure 1) was its logic and resemblance with how physiotherapists approach new patients in clinical care (Figure 2). When a new patient consults a physiotherapist (Figure 2), the physiotherapist collects information about the patients’ symptoms, performs a clinical examination, and then tries to recall his experiences with similar patients from the past (ie, *Retrieve* in Figure 2). Knowledge and experience with previous similar patients with a successful outcome are used to guide treatment for the new patient (ie, *Reuse* in Figure 2), and the treatment is adapted and revised if necessary to fit the new patient. The physiotherapist gains experience with the new patient and may thus increase their knowledge of treatment leading to a successful outcome (ie, *Retain* in Figure 2). This process resembles the structure of the CBR cycle. The main difference in problem-solving between a physiotherapist and a CBR system is that the physiotherapist is limited by his memory and experiences, while a CBR system can use experiences from many different physiotherapists and thus use a much larger case base for decision support.

### Development of the CBR System, SupportPrim PT, in Musculoskeletal Pain

In this study, we focus on the retrieval phase of CBR. We demonstrate the CBR system, SupportPrim PT, for musculoskeletal pain in two steps (1) how similar patients were identified and (2) an evaluation of SupportPrim PT’s ability to identify similar patients. The system also displays solutions (ie, treatment suggestions) for new patients based on previous successful cases, but this part will only be described briefly. The medical community is the target audience for this study, and we have, therefore, used nontechnical language.

### Patient Similarity Measures

The SupportPrim PT was built using myCBR (myCBR v3 and its rest API v2), which leverages patient data from the past to identify the most similar patients to advise management [24]. We used the local-global principle in CBR to identify similar patients, where similarity is calculated by a weighted sum function [25]. Global similarity measures are attributes used to identify similar patients, where an example of an attribute is a patient’s age or pain intensity. The weighting of these attributes (ie, global weighting) represents the relevance of the different attributes for the identification of similar patients, in our case, in terms of prognostic importance and choice of treatment, while local measures weight similarities between different values for the same attribute.

We chose attributes based on their prognostic value in previous studies of patients with musculoskeletal pain and from systematic reviews of generic prognostic factors across body regions (Multimedia Appendix 1 [26-41]). These prognostic factors inform the likely course of musculoskeletal pain to aid examination or treatment decisions [42-46]. The domains covered were sociodemographic factors, pain and function, psychological factors, and health behavior (refer to Multimedia Appendix 1 for a detailed list). In addition, some of the attributes were chosen for their potential to influence specific treatment decisions and not for their prognostic abilities, for example, physical activity and BMI for overweight [47,48].

For the development of global weights, we first created a baseline CBR system by assigning equal weights to all the attributes, a second system with assigned weights based on a data-driven approach [49], and then a CBR system that used expert knowledge. We decided to use the expert knowledge approach to emphasize evidence of prognostic factors across different musculoskeletal conditions. We validated the weighting of the attributes in an iterative process using a sample of 14 patients representing 5 distinct phenotypes of musculoskeletal complaints [50], ranging from good to poor prognosis for a successful outcome [51]. The validation aimed to retrieve the most similar patient from the same phenotype as the queried patient, which is the new patient. In addition, we weighted attributes we believed were important for choosing adequate physiotherapy treatment higher (eg, mental distress, insomnia, and work ability; Multimedia Appendix 1).

For the local similarity measures, we decided the degree of similarity between values within each attribute from 0 (not similar at all) to 1 (full conformity between scores; Multimedia Appendix 1). To guide this work, we used knowledge about minimal clinically important difference, which is the smallest difference that is clinically important for the patient. For instance, this could be 2 points in the numeric rating scale for pain intensity [52], which means that values within this range were regarded as completely similar. For attributes where information about minimal clinically important difference were lacking, we determined this by consensus within the study group. We did not always assume a linear relationship between scores on an attribute in the local similarity measure, where the local similarity for the same absolute difference in score could differ if the score was at the upper or lower end of the scale (eg, physical activity, where we defined full conformity—1.0 similar—between 5 and 6 to 7 days, while 0 versus 1 day were defined as only 0.4 similar).

### How to Find a Similar Patient

The SupportPrim PT calculates a similarity score to find similar patients. A similarity score is the weighted sum of all the local similarity scores divided by the total possible weighting (Table 1), giving a similarity score between 0 and 1. Calculation of the similarity score between a query patient (Q) and the most similar patient in the case base (C) is shown in equation 1, earlier described by Bergman [25], where “w” is the weight of the attribute “i.” For each attribute “i,” the local similarity is defined as “sim_i_ (q, c),” where “q” is the value of the attribute for the query patient and “c” is the value of that respective attribute for the patient case from the case base. Finding the most similar patient is the result of the retrieval process where all patients are compared to the query patient, and the most similar patients are returned. The patient with the highest similarity score will be the most similar to the query patient.

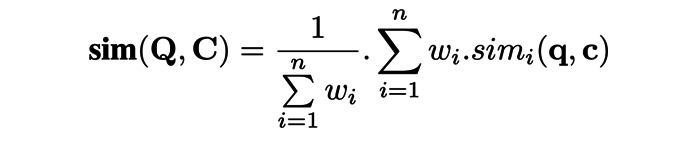



Equation 1 shows the calculation of the similarity score between a query patient (Q) and the most similar patient in the case base (C).

**Table 1:** Example of a calculation of similarity score showing the query patient with the 4 most similar patients in the case-based reasoning system SupportPrim PT for patients with musculoskeletal pain disorders.

Patient	Attributes^a^ (weight^b^)																
	Mental distress (8)		Expectations (4)			Pain sites (2)			Sleep (4)			Workability (4)			Total score		Similarity score^c^
	Score	Local sim^d^	Score	Local sim^d^	Score		Local sim^d^	Score		Local sim^d^	Score		Local sim^d^				
Query patient	2	N/A^e^	8	N/A	3		N/A	Moderate		N/A	6		N/A	N/A		N/A	
Patient A	1.8	0.8	7	1.0	3		1.0	Moderate		1.0	5		1.0	20.4		0.93	
Patient B	2.0	1.0	6	0.8	2		0.8	Great		0.6	7		1.0	19.2		0.87	
Patient C	1.5	0.6	9	1.0	4		0.8	Slight		0.8	3		0.6	16.0		0.73	
Patient D	2.2	0.8	4	0.3	5		0.6	Normal		0.4	4		0.8	13,6		0.62	

^a^The attributes used to identify similar patients.

^b^The relevance of the different attributes for the identification of similar patients, ranging from 1 to 8, where higher weights represent higher relevance.

^c^Calculation: ((attribute 1 weight×attribute 1 local sim)+(attribute 2 weight×attribute 2 local sim)+...+[attribute n weight×attribute n local sim])/(attribute 1 weight+attribute 2 weights+...+attribute n weight)=Similarity score. Patient A: [(8×0.8)+(4×1.0)+(2×1.0)+(4×1.0)+(4×1.0)]/(8+4+2+4+4)=0.93.

^d^The degree of similarity between values within each attribute. Ranges from 0 to 1, where 0 means not similar at all and 1 means full conformity between scores.

^e^N/A: not applicable.

In Table 1, we show a query patient with its 4 most similar patients ranked according to the similarity score (patient A, B, C, and D). We exemplify the calculations of the similarity score by showing 5 of the 29 global measures (ie, attributes) used to represent cases in the CBR system. Patient A will be the most similar, B second most similar, C third most similar, and D the fourth most similar. Furthermore, which is beyond the scope of this study, important patient information and treatment description from the most similar patients having successful outcome will be displayed for the physiotherapist in a clinical dashboard.

To populate the case base for the SupportPrim PT, we systematically collected data from patients and physiotherapists in primary care of Norway. The case base consists of data on patient characteristics, prognostic factors, description of treatments, and outcomes from patients aged ≥18 years with musculoskeletal pain in any of these areas: shoulder, neck, upper or low back, hip, knee, or with complex pain as primary contact reason. Classification of complex pain was at the discretion of the treating physiotherapist based on a combination of the overall severity of symptoms, the number of pain sites, the clinical examination, and the patient history.

### System’s Ability to Identify Similar Patients

To explore how the SupportPrim PT performs in finding similar patients, we assessed the similarity score of all patients in the case base, in ranked order from the most to the least similar patients, with the scores on the short form Örebro Musculoskeletal Pain Screening Questionnaire (ÖMSPQ) [53] and the Musculoskeletal Health Questionnaire (MSK-HQ) [54]. For similarity scores, each patient was compared with all other patients in the case base and repeated similarly for all patients to attain a rank order of most similar patients for all patients in the case base. Both questionnaires are used across different musculoskeletal pain conditions. ÖMSPQ is an established prognostic tool for long-term disability and failure of return to work, with a total score of 0 to 100, where higher scores indicate a worse long-term disability. The ÖMSPQ questionnaire emphasizes biopsychosocial variables related to future disability—similar to the global measures in the CBR system. The ÖMSPQ includes pain, self-perceived function, distress, return to work expectancy, and fear avoidance beliefs. The MSK-HQ is a generic musculoskeletal outcome measure that can be used for different musculoskeletal conditions. It contains 14 key items: severity of pain or stiffness, physical function or activity, work or daily activities, symptoms interference, independence, sleep, fatigue or low-energy levels, emotional well-being, understanding of condition and treatment, confidence in being able to manage symptoms, and overall symptom impact. The total score range is 0 to 56, with higher scores indicating better musculoskeletal health.

Assessing the similarity scores with the 2 established instruments was done with the case base used to build the SupportPrim PT (n=105) and then repeated in a larger case base (n=486). For the latter, we imported additional patients in the case base from another study to assess the performance of the similarity scores in a larger case base.

For patients with musculoskeletal problems, there is rarely 1 ideal solution. Different treatments could lead to a satisfactory outcome and, thus, work as solutions to the problem. In the final CBR system, after identifying similar patients using the local-global principle, similar previous successful patients were filtered, and the description of their treatment was displayed to inform treatment for the new patient (“solution”). The criterion for a successful outcome was a combination of pain intensity and function measured at baseline and at 3-month follow-up, where also the change score on MSK-HQ and the patient’s global perceived effect were included in the combined outcome measure. Details of our definition of a successful outcome are described in Multimedia Appendix 2 [53-56].

### Ethical Considerations

The Regional Committee for Medical and Health Research Ethics in mid-Norway approved the study (51566/2019 and 49308/2020). All patients provided written informed consent to participate in the study. Patients did not receive any compensation for participating. Study data are deidentified, and no identification of individual participants is possible.

## Results

### Descriptive Characteristics of Patients in the Case Base

The original case base consisted of 105 patients, with complete data gathered from 22 physiotherapists in primary care collected from January 2020 to January 2021. The patients’ mean age was 46 (SD 15) years, the majority were women (77/105, 73.3%), and pain duration was >3 months for most patients (87/105, 82.9%; Table 2).

**Table 2:** Characteristics of patients with musculoskeletal pain disorders included in the case-based reasoning system SupportPrim PT (N=105).

		Total cohort
Female, n (%)		77 (73.3)
Age (years), mean (SD)		46.0 (15.2)
BMI (kg/m^2^), mean (SD)		26.9 (6.1)
Higher education^a^, n (%)		59 (56.2)
Pain duration^b^, n (%)		87 (82.9)
Current smoker, n (%)		9 (8.6)
Pain intensity, mean (SD)		4.7 (2.1)
PSFS^c^, mean (SD)		4.0 (2.7)
Work ability, mean (SD)		6.2 (2.9)
**Musculoskeletal risk group^d^ (n=101), n (%)**		
	Low	42 (41.6)
	Medium	47 (46.5)
	High	12 (11.9)
**Main body pain region, n (%)**		
	Neck	16 (15.2)
	Shoulder	18 (17.1)
	Back	19 (18.1)
	Hip	16 (15.2)
	Knee	13 (12.4)
	Complex	23 (21.9)
MSK-HQ^e^, mean (SD)		37.7 (8.5)
ÖMSPQ^f^, mean (SD)		43.1 (15.6)

^a^Education above high school.

^b^Pain duration >3 months.

^c^PSFS: Patient Specific Functional Scale; a higher value indicates better function.

^d^Musculoskeletal risk groups: 0-4 is low risk, 5-8 is medium risk, and 9-12 is high risk.

^e^MSK-HQ: Musculoskeletal Health Questionnaire; a higher value indicates better musculoskeletal health.

^f^OMSPQ: Örebro Musculoskeletal Pain Screening Questionnaire; higher scores indicate worse long-term disability.

### The System’s Ability to Identify Similar Patients

**Figure 3:**
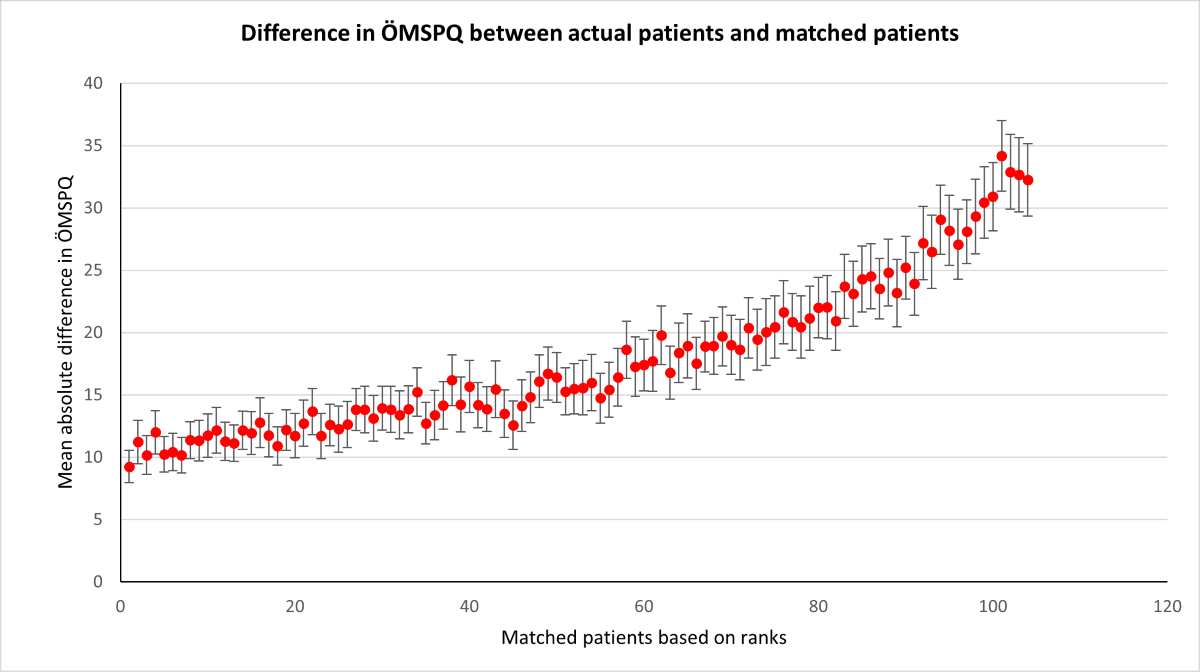
The absolute difference (mean with 95% CI) in the short form Örebro Musculoskeletal Pain Screening Questionnaire (ÖMSPQ) between queried patients with musculoskeletal pain disorders and most similar patients in ranked order in the case-based reasoning system SupportPrim PT.

**Figure 4:**
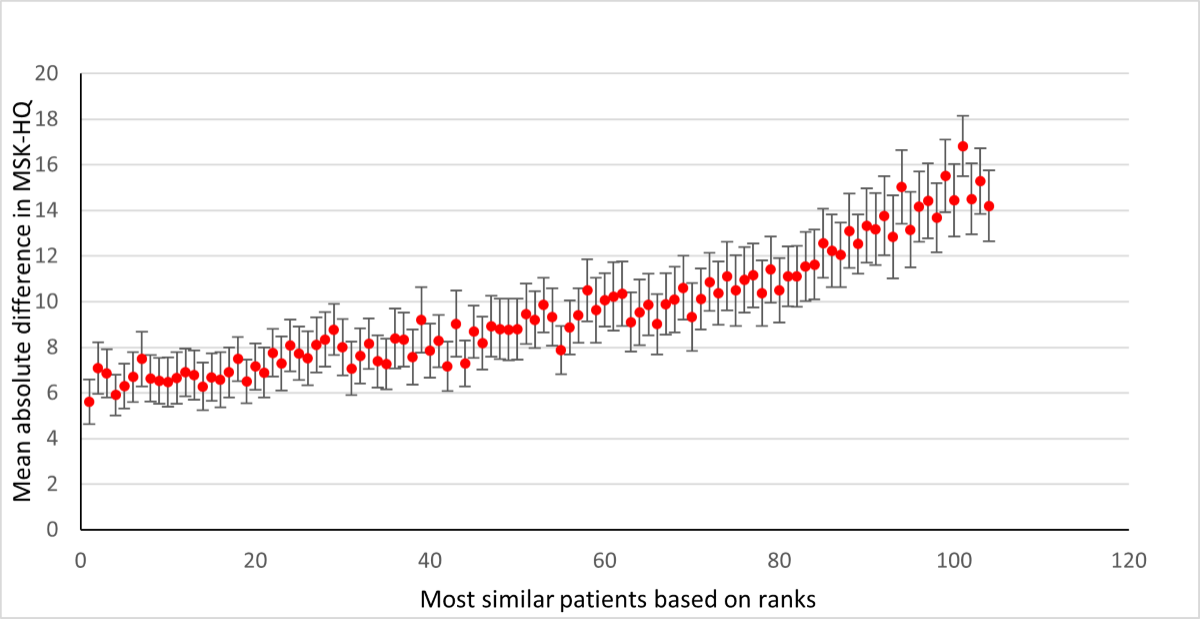
The absolute difference (mean with 95% CI) in the Musculoskeletal Health Questionnaire (MSK-HQ) between queried patients with musculoskeletal pain disorders and most similar patients in ranked order in the case-based reasoning system SupportPrim PT.

The SupportPrim PT built to use for decision support in patients with musculoskeletal pain consisted of 29 weighted attributes, each having a defined local similarity measure to identify similar patients. To demonstrate the system’s ability to identify the most similar patients, all patients in the case base were compared to each other, that is, each patient was queried against the rest of the patients, and this was repeated for all patients. Rank 1 thus represents the average similarity score for the most similar patient (“best match”) to the query patient for all patients in the case base. The ranks from the most similar to least similar were then plotted against each rank’s absolute difference on the ÖMSPQ (Figure 3) and MSK-HQ (Figure 4) scores. The most similar patients had a mean absolute difference from the query patient of 9.3 (95% CI 8.0-10.6) points on the ÖMSPQ and a mean absolute difference of 5.6 (95% CI 4.6-6.6) points on the MSK-HQ. For both ÖMSPQ and MSK-HQ, the absolute score difference increased as the rank of most similar patients decreased (Figures 3 and 4).

**Figure 5:**
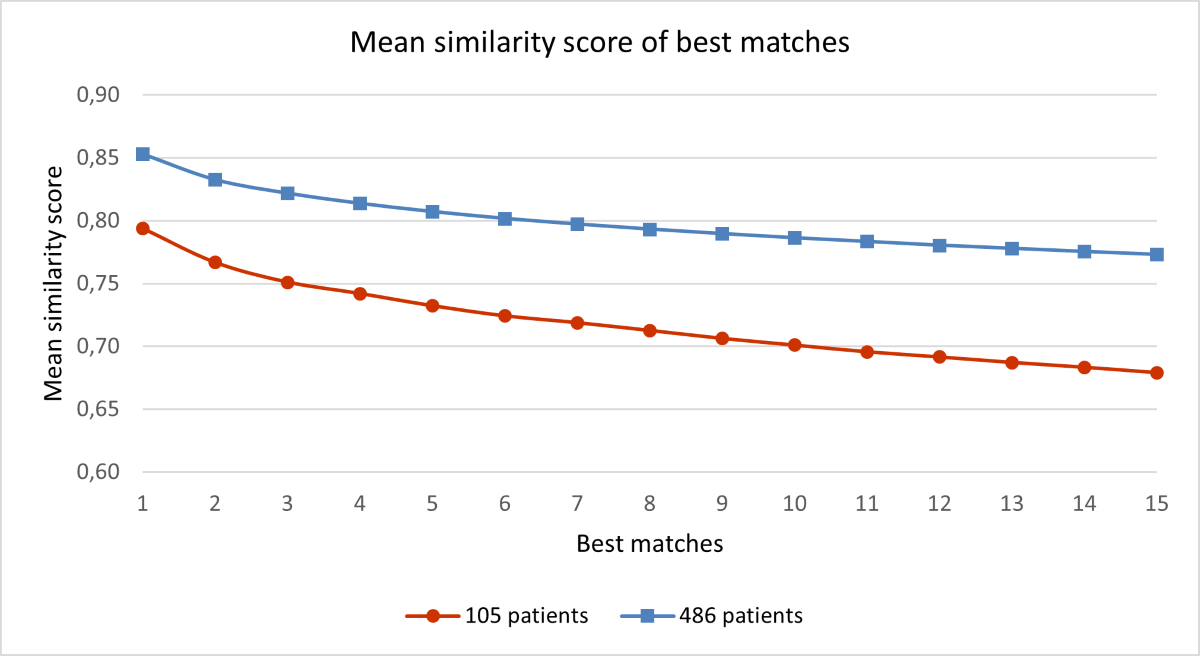
The mean similarity score of the 15 most similar patients with musculoskeletal pain disorders in the 2 case bases in the case-based reasoning system SupportPrim PT.

**Figure 6:**
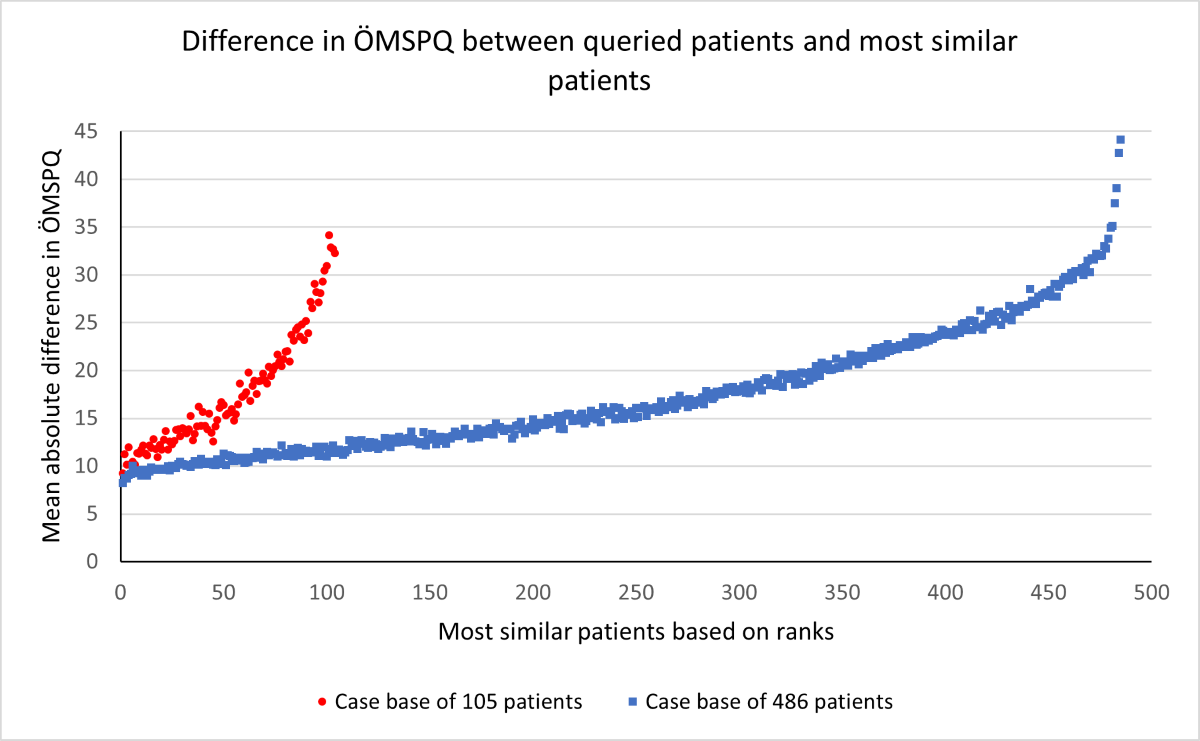
The absolute mean difference in the short form Örebro Musculoskeletal Pain Screening Questionnaire (ÖMSPQ) between queried patients with musculoskeletal pain disorders and most similar patients in ranked order for the 2 different size case bases in the case-based reasoning system SupportPrim PT.

**Figure 7:**
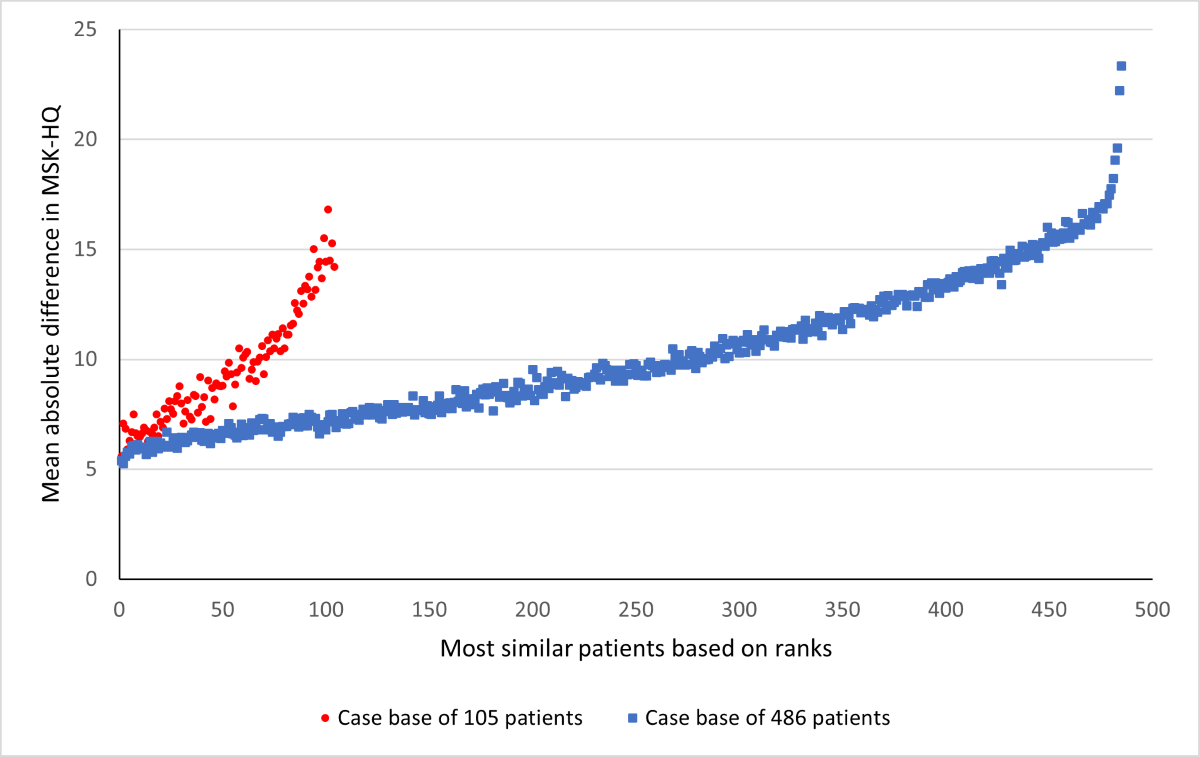
The absolute mean difference in the Musculoskeletal Health Questionnaire (MSK-HQ) between the queried patients with musculoskeletal pain disorders and the most similar patients in ranked order for the 2 different size case bases in the case-based reasoning system SupportPrim PT.

To assess the performance of the similarity scores in a larger case base, we imported additional patients into the case base, resulting in a case base of 486 patients. When we compare the mean similarity score for the most similar patients for the 2 case bases, we see that the patients retrieved from the larger case base had a slightly higher mean similarity score (Figure 5). We compared the ranks of most similar patients for all patients in both case bases with the ÖMSPQ (Figure 6 and Table 3) and MSK-HQ (Figure 7 and Table 4). The results showed that the larger case base identified slightly more similar patients with a smaller absolute mean difference on ÖMSPQ and MSK-HQ for the ranks compared to the original case base.

**Table 3:** The absolute mean difference in the short form Örebro Musculoskeletal Pain Screening Questionnaire (score range 0-100) between queried patients with musculoskeletal pain disorders and the 3 most similar patients for the 2 different case bases in the case-based reasoning system SupportPrim PT.

	Case base with 105 patients, absolute mean difference (95% CI)	Case base with 486 patients, absolute mean difference (95% CI)
Most similar patient	9.3 (8.0-10.6)	8.2 (7.7-8.7)
Second most similar patient	11.2 (9.5-13.0)	8.8 (8.2-9.4)
Third most similar patient	10.2 (8.6-11.7)	8.7 (8.1-9.3)

**Table 4:** The absolute mean difference in the Musculoskeletal Health Questionnaire (score range 0-56) between queried patients with musculoskeletal pain disorders and the 3 most similar patients for the 2 different case bases in the case-based reasoning system SupportPrim PT.

	Case base with 105 patients, absolute mean difference (95% CI)	Case base with 486 patients, absolute mean difference (95% CI)
Most similar patient	5.6 (4.6-6.6)	5.4 (5.0-5.7)
Second most similar patient	7.1 (6.0-8.2)	5.3 (4.9-5.6)
Third most similar patient	6.8 (5.8-7.9)	5.6 (5.2-6.0)

## Discussion

### Principal Findings

This study describes the development of the CBR system, SupportPrim PT, using the local-global principle to identify similar patients with musculoskeletal pain disorders. The SupportPrim PT successfully identified similar patients.

When comparing the similarity scores from the SupportPrim PT and their rankings with ÖMSPQ and MSK-HQ scores, we found that the SupportPrim PT successfully identified the most similar patients to the queried patients across the musculoskeletal pain conditions (Figures 3 and 4). The mean score differences on the questionnaires between the queried and the most similar patients increased linearly or curvilinearly with increasing rank order (less similar patients). These results are not unexpected as SupportPrim PT contains 29 mainly prognostic attributes partly overlapping with items on the ÖMSPQ and MSK-HQ. Mean differences between queried patients and best matches of 9.3 points on ÖMSPQ and 5.6 points on the MSK-HQ are also expected. The SupportPrim PT uses a larger number of attributes for patient similarity comparisons, possibly making the system more comprehensive in mapping patients’ symptoms and prognostic factors than the shorter comparative questionnaires. Using a larger case base, the SupportPrim PT yielded patients with slightly higher mean similarity scores and lower absolute mean difference between the queried patients and the most similar patients on the ÖMSPQ and MSK-HQ. This may indicate that a larger case base may improve the performance of the system [57]. The case base of 105 patients is nevertheless representative of primary physiotherapy care in Norway, with descriptive data being consistent with data from a large longitudinal observational study [58].

Key challenges when developing a CBR system are definition of the case representation, similarity measure development, and retrieval strategy. Attribute selection and weighting could be dependent on expert knowledge and limited by the number, type, and quality of the attributes included. We selected attributes based on their prognostic value or potential to influence treatment choice. There is good documentation for prognostic factors being similar across musculoskeletal diagnostic groups [42-46]. Classifying patients according to similar prognostic factors rather than diagnosis may be more fruitful in improving care [9]. To base interventions on diagnoses is relevant if a causal pathway between diagnosis and choice of treatment is established. However, a clear understanding of causal pathways is often lacking in musculoskeletal pain complaints. Thus, physiotherapy interventions are commonly directed toward symptom alleviation, for example, advice, reassurance, self-management, exercise, and manual therapy. Therefore, patients may best be treated within a prognostic framework with more emphasis on specific prognostic factors on the individual level [43].

Definition of global weights in CBR is challenging. We used expert knowledge instead of data-driven methods [49]. Among the attributes selected, the sum score of Hopkins Symptom Checklist 10-item, with questions about anxiety, depression, and somatization, was weighted highest, as emotional distress is an important mediating factor for the treatment effect [59] and also potentially modifiable by physiotherapy interventions [60]. Decisions were based on consensus between the authors with different backgrounds and extensive experience both from research and clinical work.

### Limitations

Instead of attribute selection based on expert knowledge, automated data-driven attribute selection methods [61] could have been used. We acknowledge that other attributes not included in our system could have improved the process of identifying similar patients. A limitation of the study is that the process of assigning weights of the global measures did not follow a formal consensus method. This could have resulted in different weighting of the attributes. In addition, comparative studies of data-driven and expert-driven approaches to decide the weighting of different attributes should be explored in future work.

### Conclusions

Advising treatment for new patients using previous similar patients with successful outcome represents a move toward more individualized treatment rather than relying on the best evidence of average effects in clinical trials. It is important to acknowledge that the SupportPrim PT is not built to replace the clinical expertise and experience of the therapist but to work as a decision support. Furthermore, AI might create an uncomfortable situation for clinicians and patients, not having complete control and being uncertain, not understanding what is in the system, not having the possibility to tell which attributes are used in the model, and thus not trusting the system [10]. We believe CBR can address this uncertainty by being an easy-to-understand and explainable AI method [62], with expert and domain knowledge being an integrated part of the system, which increases the likelihood for clinicians to trust it. This study describes the development of a CBR system, SupportPrim PT, for musculoskeletal pain in primary care. It demonstrates the system’s ability to identify similar patients on an established screening tool and an outcome measure used for patients with musculoskeletal pain disorders. The SupportPrim PT will be integrated into a clinical decision support system and tested in a full-scale randomized controlled trial in primary health care to evaluate its effectiveness among physiotherapists and their patients. The SupportPrim PT was developed for decision support for physiotherapists in managing patients with musculoskeletal pain disorders, but we think such an explainable system could be applicable to other health care personnel for patients where decision support is needed.

## Appendix

Multimedia Appendix 1 Attributes in the case-based reasoning system, SupportPrim PT.

Multimedia Appendix 2 Definition of a successful outcome in the case-based reasoning system, SupportPrim PT.

## Abbreviations

AI: artificial intelligence
CBR: case-based reasoning
MSK-HQ: Musculoskeletal Health Questionnaire
ÖMSPQ: Örebro Musculoskeletal Pain Screening Questionnaire

## Data Availability

The data sets generated and analyzed during this study are available from the corresponding author on reasonable request.
