# Supplementary material for: Decision Support for Managing Common Musculoskeletal Pain Disorders: Development of a Case-Based Reasoning Application
Source: JMIR Form Res. 2024 May 10;8:e44805. doi: 10.2196/44805 (PMC11127158; doi:10.2196/44805)
Supplement: Multimedia Appendix 1 [file formative_v8i1e44805_app1.docx]

**Multmedia appendix 1.** Attributes in the case-based reasoning system, SupportPrim PT, for musculoskeletal pain disorders.

| Global similarity measures^a^ | | | | | Local similarity measures^b^ |
| --- | --- | --- | --- | --- | --- |
| Attributes (relevance for prognosis or choice of treatment) | | Description (reference for questionnaire or item) | Response options/scale | Weights^c^ | Local similarities |
| **Sociodemographic factors** | | | | | |
|  | Age (1, 2) | Age in years | Continuous scale | 1 | 0-5 y difference=1.0 similar.  5-10 y difference=0.8 similar.  10-15 y difference=0.6 similar.  15-20 y difference=0.4 similar.  20-30 y difference=0.2 similar. |
|  | Sex (3) | —^d^ | Binary: female or male | 1 | Same sex=1.0 similar.  Otherwise not similar. |
|  | Education (4, 5) | What is your highest level of education? | Ordered list: primary school or less, high school, 4 y of higher education or less, and >4 y of higher education | 2 | Same answer=1.0 similar.  ≤4 y to >4 y=0.6 similar.  Primary school vs high school=0.4 similar.  High school vs 4 y of higher education or less=0.4 similar. |
|  | Co-morbidities (6) | Other current illnesses or ailments | The number of comorbidities is grouped into 4 categories: 0, 1, 2-3, and ≥4 | 1 | Same answer=1.0 similar.  The category next to the actual=0.6 similar.  The category second next to the actual=0.2 similar. |
|  | Employment: 3 different options (3) | What is your work situation now? | Categorized into 3 groups: sick leave; work assessment allowance or disability pension; and in paid work, student, pensioner, nonpaid work, unemployed, or parental leave | 1 | Same answer=1.0 similar.  Sick leave vs work assessment allowance, or disability pension=0.6 similar. |
|  | Social activities and hobbies (2, 3, 7) | The MSK-HQ^e^ item 7: “How much have your joint or muscle symptoms interfered with your social activities and hobbies in the last two weeks?” (8) | “Not at all,” “Slightly,” “Moderately,” “Severely,” and “Extremely” | 1 | Same answer=1.0 similar.  Not at all vs slightly=0.8 similar.  Slightly vs moderately=0.8 similar.  Severely vs extremely=0.8 similar.  Moderately vs severely=0.6 similar.  Not at all vs moderately=0.4 similar.  Slightly vs severely=0.2 similar. Moderately vs extremely=0.2 similar. |
|  | Type of work (1) | How would you describe your work (paid or unpaid)? | “Mostly seated,” “Much walking,” “Much walking and lifting,” and “Heavy work using the body” | 2 | Same answer=1.0 similar.  Much walking and lifting vs heavy work using the body=0.8 similar.  Much walking vs much walking and lifting=0.6 similar.  Mostly seated vs much walking=0.4 similar.  Mostly seated vs much walking and lifting=0.2 similar.  Much walking and lifting vs heavy work using the body=0.2 similar. |
| **Pain and function** | | | | | |
|  | Number of pain sites (2, 7, 9) | Indicate affected body areas on a body figure: head, neck, shoulders, upper back, lower back, elbows, wrists or hands, hip or thigh, knee, and ankle or feet (10) | 11-point numerical scale (0=no pain sites and 10=10 pain sites) | 2 | Null points difference=1.0 similar.  1 point difference=0.8 similar.  2 points difference=0.6 similar.  3 points difference=0.3 similar  4 points difference=0.1 similar |
|  | Main problem | The patient’s body region group classified by the therapist | Neck, shoulder, back, hip, knee, and complex | 4 | Same body region=1.0 similar.  Otherwise not similar. |
|  | Continuous pain or not (3, 7) | “Is the pain continuous?” | “Yes, I can feel it all the time” and “No, it is on and off” | 1 | Same answer=1.0 similar.  Otherwise not similar. |
|  | Pain intensity (2, 7, 9) | Örebro Musculoskeletal Pain Screening Questionnaire item 2: “How would you rate the pain that you have had during the past week?” (11) | 11-point numerical scale (0=no pain and 10=pain as bad as it could be) | 1 | 0-1 points difference=1.0 similar.  2 points difference=0.8 similar.  3 points difference=0.6 similar.  4 points difference=0.3 similar.  5 points difference=0.1 similar. |
|  | Walking aids (2, 3, 7) | Do you use walking aids? | “Yes” or “No” | 4 | Same answer=1.0 similar.  Otherwise not similar |
|  | Pain duration (2, 3, 7) | Örebro Musculoskeletal Pain Screening Questionnaire item 1: “How long have you had your current complaints?” (11) | 10 response options range from <1 wk to >1 y. On the basis of these options, we constructed a new attribute: <4 wk, between 4 and 12 wk, between 3 and 6 mo, between 6 and 12 mo, and >1 y | 4 | Same answer=1.0 similar.  The category next to the actual=0.8 similar.  The category second next to the actual=0.4 similar. |
|  | Mobility (2, 3, 7) | Health-related quality of life questionnaire (EQ-5D^f^), item about mobility (12) | “I have no problems in walking about,” “I have slight problems in walking about,” “I have moderate problems in walking about,” “I have severe problems in walking about,” and “I am unable to walk about” | 1 | Same answer=1.0 similar.  No vs slight=0.8 similar.  Slight vs moderate=0.8 similar. Severe vs unable=0.8 similar.  Moderate vs severe=0.6 similar.  No vs moderate=0.4 similar.  Slight vs severe=0.2 similar. Moderate vs unable=0.2 similar. |
|  | Work ability (13) | “What is your current work ability compared with the lifetime best?” (14) | 11-point numerical scale (0=cannot work and 10=working at best) | 4 | 0-1 points difference=1.0 similar.  2 points difference=0.8 similar.  3 points difference=0.6 similar.  4 points difference=0.3 similar.  5 points difference=0.1 similar. |
|  | Personal care (3) | Health-related quality of life questionnaire (EQ-5D), item about care (12) | “I have no problems washing or dressing myself,” “I have slight problems washing or dressing myself,” “I have moderate problems washing or dressing myself,” “I have severe problems washing or dressing myself,” and “I am unable to wash or dress myself.” | 1 | Same answer=1.0 similar.  No vs slight=0.8 similar.  Slight vs moderate=0.8 similar. Severe vs unable=0.8 similar.  Moderate vs severe=0.6 similar.  No vs moderate=0.4 similar.  Slight vs severe=0.2 similar.  Moderate vs unable=0.2 similar. |
|  | Daily activity level (2, 3, 7) | “Due to pain or complaints, how much reduced are your activities of daily life?” | “Very much reduced,” “Quite reduced,” “Slightly reduced,” and “Not reduced” | 2 | Same answer=1.0 similar.  The category next to the actual=0.6 similar.  The category second next to the actual=0.2 similar. |
| **Psychological factors** | | | | | |
|  | Mental distress (1, 3, 7) | The Hopkins Symptom Checklist-10 item (15, 16) | Mean score across the 10 items ranging from 1.00 to 4.00 (higher scores indicate higher levels of mental distress) | 8 | 0-0.1 points difference=1.0 similar.  0.2 points difference=0.8 similar.  0.5 points difference=0.6 similar.  0.8 points difference=0.3 similar.  1.1 points difference=0.1 similar. |
|  | Pain self-efficacy (7, 17) | Two questions: (1) “I can do some form of work, despite pain (work includes housework and paid or unpaid work)” and (2) “I can live a normal lifestyle, despite pain” (18) | Response options ranging from 0 (not at all) to 6 (completely confident) on both questions. These response options were then collapsed, ranging from 0-12 (higher score indicate higher levels of self-efficacy). | 2 | 0-1 points difference=1.0 similar.  2 points difference=0.8 similar.  3 points difference=0.6 similar.  4 points difference=0.4 similar. |
|  | Expectations of recovery (17, 19, 20) | Örebro Musculoskeletal Pain Screening Questionnaire item 7: “In your view, how large is the risk that your current pain may become persistent?” (11) | 11-point numerical scale (0=no risk and 10=high risk) | 4 | 0-1 points difference=1.0 similar.  2 points difference=0.8 similar.  3 points difference=0.6 similar.  4 points difference=0.3 similar.  5 points difference=0.1 similar. |
|  | Sleep difficulties (3) | A single item from 15D^g^: patients were asked to define their level of sleep problems (21) | “I’m able to sleep normally,” “I have slight problems with sleeping,” “I have moderate problems with sleeping,” “I have great problems with sleeping,” and “I suffer severe sleeplessness” | 4 | Same answer=1.0 similar.  Normally vs slight=0.8 similar.  Slight vs moderate=0.8 similar.  Great vs severe=0.8 similar.  Moderate vs great=0.6 similar.  Normally vs moderate=0.4 similar.  Slight vs great=0.2 similar. Moderate vs severe=0.2 similar. |
|  | Vitality (3, 4) | Single item from the 15D instrument of health-related quality of life: patients were asked to define their level of vitality problems (21) | “I feel healthy and energetic;” “I feel slightly weary, tired, or feeble;” “I feel moderately weary, tired or feeble;” “I feel very weary, tired, or feeble, almost exhausted;” and “I feel extremely weary, tired, or feeble, totally exhausted” | 1 | Same answer=1.0 similar.  Healthy and energetic vs slightly=0.8 similar.  Slightly vs moderately=0.8 similar.  Very vs extremely=0.8 similar.  Moderately vs very=0.6 similar.  Healthy and energetic vs moderately=0.4 similar.  Slightly vs very=0.2 similar. Moderately vs extremely=0.2 similar. |
|  | Fear avoidance (3) | Örebro Musculoskeletal Pain Screening Questionnaire item 10: “I should not do my normal activities or work with my present pain?” (11) | 11-point numerical scale (0=disagree and 10=completely agree) | 1 | 0-1 points difference=1.0 similar.  2 points difference=0.8 similar.  3 points difference=0.6 similar.  4 points difference=0.3 similar.  5 points difference=0.1 similar. |
|  | Kinesio-phobia (3) | “How much fear do you have that these complaints would be increased by physical activity?” (22) | 11-point numerical scale (0=no fear and 10=high fear) | 1 | 0-1 points difference=1.0 similar.  2 points difference=0.8 similar.  3 points difference=0.6 similar.  4 points difference=0.3 similar.  5 points difference=0.1 similar. |
|  | Anxiety and depression (3, 7) | Health-related quality of life questionnaire (EQ-5D), item about anxiety and depression (12) | “I am not anxious or depressed,” “I am slightly anxious or depressed,” “I am moderately anxious or depressed,” “I am severely anxious or depressed,” and “I am extremely anxious or depressed” | 2 | Same answer=1.0 similar.  Not vs slightly=0.8 similar.  Slightly vs moderately=0.8 similar. Severely vs extremely=0.8 similar.  Moderately vs severely=0.6 similar.  Not vs moderately=0.4 similar.  Slightly vs severely=0.2 similar. Moderately vs extremely=0.2 similar. |
| **Health behavior** | | | | | |
|  | BMI | Self-reported height and weight | Calculated from weight and height reported by the patient. Measured on a continuous scale | 1 | 0-1.5 points difference=1.0 similar.  1.5-3.0 points difference=0.7 similar.  3.0-4.5 points difference=0.4 similar. |
|  | Smoking (3) | “Do you smoke?” | “Yes” or “no” | 2 | Same answer=1.0 similar.  Otherwise not similar. |
|  | Physical activity | The MSK-HQ activity item: “In the past week, on how many days have you done a total of 30 minutes or more of physical activity, which was enough to raise your heart rate?” (8) | 8 answering options, 0-7 d | 2 | Same number of days=1.0 similar.  3 vs 4 d=1.0 similar.  4 vs (5 to 6) d=1.0 similar.  5 vs (6 to 7) d=1.0 similar.  2 vs 3 d=0.8 similar.  4 vs 7 d=0.8 similar.  1 vs 2 d=0.6 similar.  2 vs 4 d=0.6 similar.  3 vs (5 to 7) d=0.6 similar.  0 vs 1 d=0.4 similar.  0 vs 2 d=0.2 similar.  1 vs 3 d=0.2 similar.  2 vs (5 to 7) d=0.2 similar. |
| **Risk group** | | | | | |
|  | Risk group | The Keele STarT MSK Tool (23) | Contains 10 items that place patients into 3 categories based on their risk of poor outcome (low, medium, and high) | 4 | Same category=1.0 similar.  The category next to the actual one=0.2 similar. |

^a^Global similarity measures represent the attributes used to identify similar patients.

^b^Local similarity measures represent the degree of similarity between values within each attribute, ranging from 0 to 1, where 0 means not similar at all and 1 means full conformity between scores.

^c^Weights represent the relevance of the different attributes for the identification of similar patients, ranging from 1 to 8, where higher weights represent higher relevance.

^d^Not applicable.

^e^MSK-HQ: Musculoskeletal Health Questionnaire.

^f^EQ-5D: EuroQol 5 dimensions.

^g^15D: Instrument of health-related quality of life.

1. Green DJ, Lewis M, Mansell G, Artus M, Dziedzic KS, Hay EM, et al. Clinical course and prognostic factors across different musculoskeletal pain sites: A secondary analysis of individual patient data from randomised clinical trials. European Journal of Pain. 2018;22(6):1057-70. https://doi.org/10.1002/ejp.1190

2. Valentin GH, Pilegaard MS, Vaegter HB, Rosendal M, Ørtenblad L, Væggemose U, et al. Prognostic factors for disability and sick leave in patients with subacute non-malignant pain: a systematic review of cohort studies. BMJ Open. 2016;6(1):e007616. http://doi.org/10.1136/bmjopen-2015-007616

3. de Vos Andersen NB, Kent P, Hjort J, Christiansen DH. Clinical course and prognosis of musculoskeletal pain in patients referred for physiotherapy: does pain site matter? BMC Musculoskelet Disord. 2017;18(1):130. http://doi.org/10.1186/s12891-017-1487-3

4. Henschke N, Ostelo RWJG, Terwee CB, van der Windt DAWM. Identifying generic predictors of outcome in patients presenting to primary care with nonspinal musculoskeletal pain. Arthritis Care Res. 2012;64(8):1217-24. https://doi.org/10.1002/acr.21665

5. Nahit ES, Hunt IM, Lunt M, Dunn G, Silman AJ, Macfarlane GJ. Effects of psychosocial and individual psychological factors on the onset of musculoskeletal pain: common and site-specific effects. Annals of the rheumatic diseases. 2003;62(8):755-60. http://doi.org/10.1136/ard.62.8.755

6. Vigdal ØN, Storheim K, Killingmo RM, Småstuen MC, Grotle M. The one-year clinical course of back-related disability and the prognostic value of comorbidity among older adults with back pain in primary care. Pain. 2022. https://doi.org/10.1097/j.pain.0000000000002779

7. Artus M, Campbell P, Mallen CD, Dunn KM, van der Windt DAW. Generic prognostic factors for musculoskeletal pain in primary care: a systematic review. BMJ Open. 2017;7(1):e012901. https://doi.org/10.1136/bmjopen-2016-012901

8. Hill JC, Kang S, Benedetto E, Myers H, Blackburn S, Smith S, et al. Development and initial cohort validation of the Arthritis Research UK Musculoskeletal Health Questionnaire (MSK-HQ) for use across musculoskeletal care pathways. BMJ Open. 2016;6(8):e012331. http://doi.org/10.1136/bmjopen-2016-012331

9. Hartvigsen J, Hancock MJ, Kongsted A, Louw Q, Ferreira ML, Genevay S, et al. What low back pain is and why we need to pay attention. The Lancet. 2018;391(10137):2356-67. https://doi.org/10.1016/S0140-6736(18)30480-X

10. Kuorinka I, Jonsson B, Kilbom A, Vinterberg H, Biering-Sørensen F, Andersson G, et al. Standardised Nordic questionnaires for the analysis of musculoskeletal symptoms. Appl Ergon. 1987;18(3):233-7. https://doi.org/10.1016/0003-6870(87)90010-X

11. Linton SJ, Nicholas M, MacDonald S. Development of a Short Form of the Örebro Musculoskeletal Pain Screening Questionnaire. Spine (Phila Pa 1976). 2011;36(22). https://doi.org/10.1097/brs.0b013e3181f8f775

12. Solberg TK, Olsen JA, Ingebrigtsen T, Hofoss D, Nygaard OP. Health-related quality of life assessment by the EuroQol-5D can provide cost-utility data in the field of low-back surgery. European spine journal : official publication of the European Spine Society, the European Spinal Deformity Society, and the European Section of the Cervical Spine Research Society. 2005;14(10):1000-7. http://doi.org/10.1007/s00586-005-0898-2

13. Lagersted-Olsen J, Bay H, Jørgensen MB, Holtermann A, Søgaard K. Low back pain patterns over one year among 842 workers in the DPhacto study and predictors for chronicity based on repetitive measurements. BMC Musculoskeletal Disorders. 2016;17(1):453. http://doi.org/10.1186/s12891-016-1307-1

14. El Fassi M, Bocquet V, Majery N, Lair ML, Couffignal S, Mairiaux P. Work ability assessment in a worker population: comparison and determinants of Work Ability Index and Work Ability score. BMC Public Health. 2013;13(1):305. http://doi.org/10.1186/1471-2458-13-305

15. Derogatis LR, Lipman RS, Rickels K, Uhlenhuth EH, Covi L. The Hopkins Symptom Checklist (HSCL). A measure of primary symptom dimensions. Modern problems of pharmacopsychiatry. 1974;7(0):79-110. https://doi.org/10.1159/000395070

16. Strand BH, Dalgard OS, Tambs K, Rognerud M. Measuring the mental health status of the Norwegian population: A comparison of the instruments SCL-25, SCL-10, SCL-5 and MHI-5 (SF-36). Nordic journal of psychiatry. 2003;57(2):113-8. http://doi.org/10.1080/08039480310000932

17. Chester R, Jerosch-Herold C, Lewis J, Shepstone L. Psychological factors are associated with the outcome of physiotherapy for people with shoulder pain: a multicentre longitudinal cohort study. Br J Sports Med. 2016. http://doi.org/10.1136/bjsports-2016-096084

18. Nicholas MK, McGuire BE, Asghari A. A 2-Item Short Form of the Pain Self-Efficacy Questionnaire: Development and Psychometric Evaluation of PSEQ-2. The Journal of Pain. 2015;16(2):153-63. https://doi.org/10.1016/j.jpain.2014.11.002

19. Bishop MD, Mintken P, Bialosky JE, Cleland JA. Patient Expectations of Benefit From Interventions for Neck Pain and Resulting Influence on Outcomes. Journal of Orthopaedic & Sports Physical Therapy. 2013;43(7):457-65. http://doi.org/10.2519/jospt.2013.4492

20. Foster NE, Bishop A, Thomas E, Main C, Horne R, Weinman J, et al. Illness perceptions of low back pain patients in primary care: What are they, do they change and are they associated with outcome? Pain. 2008;136(1). https://doi.org/10.1016/j.pain.2007.12.007

21. Sintonen H. The 15D instrument of health-related quality of life: properties and applications. Annals of medicine. 2001;33(5):328-36. http://doi.org/10.3109/07853890109002086

22. Verwoerd AJ, Luijsterburg PA, Timman R, Koes BW, Verhagen AP. A single question was as predictive of outcome as the Tampa Scale for Kinesiophobia in people with sciatica: an observational study. J Physiother. 2012;58(4):249-54. http://doi.org/10.1016/s1836-9553(12)70126-1

23. Dunn KM, Campbell P, Lewis M, Hill JC, van der Windt DA, Afolabi E, et al. Refinement and validation of a tool for stratifying patients with musculoskeletal pain. European Journal of Pain. 2021;25(10):2081-93. https://doi.org/10.1002/ejp.1821
