# Supplementary material for: Decision Support for Managing Common Musculoskeletal Pain Disorders: Development of a Case-Based Reasoning Application
Source: JMIR Form Res. 2024 May 10;8:e44805. doi: 10.2196/44805 (PMC11127158; doi:10.2196/44805)
Supplement: Multimedia Appendix 2 [file formative_v8i1e44805_app2.docx]

**Appendix**

This appendix describes the definition of the outcome. To catch what could be important for different patients we used different combinations of pain intensity last week, function, global perceived effect, work ability and MSK-HQ to define successful patients.

**Variables used for the outcome**

Pain intensity was assessed with the question “How would you rate the pain that you have had during the past week”, from 0 (no pain) to 10 (as bad as it could be) (1).

The Patient Specific Function Scale (PSFS) (NRS, 0-10) assessed function (2). Patients chose one activity they particularly had difficulty performing and scored the degree of difficulty on a scale from 0 (unable to perform) to 10 (able to perform at prior level). The activity was defined together with the physiotherapist at the first consultation, and at follow-up questionnaires, the participant rated the same activity again.

Work ability was measured by a single question from the Work Ability Index questionnaire: «What is your current work ability compared with the lifetime best?” scored from 0 (cannot work) to 10 (working at best) (3).

Global improvement after treatment was assessed by the Global Perceived Effect Scale (GPE). The question is phrased: “Since treatment started, I am….?”, with response options on a 7-point Likert scale from 1 (“very much improved”) to 7 (“very much worse”) with “no change” as the mid-point (4)

The Musculoskeletal Health Questionnaire (MSK-HQ) (5) is a questionnaire developed to assess outcomes in patients with a variety of musculoskeletal conditions. It is scored on a range of 0-56, with higher scores indicating better MSK health.

**Outcome**

Treatment outcome for the patients was dichotomized into success and non-success as follows:

At baseline (Table 1), the patients' scores on pain and function/disability were used to sort patients into four risk groups, reflecting their symptom pressure. Pain intensity last week (NRS; 0-10, where 0 means no pain) and function/disability (Patient-Specific Functional Scale; 0-10, where 0 means unable to perform defined activity), i.e., "best score" was in opposite direction for the two scales. Outcome at 3 months follow-up was calculated according to table 2 and governed by the patient's risk group. The rationale being that measuring effects on pain and function/disability only makes sense if they represent the patient's problem initially at baseline. Note that "non-successful" cases by these calculations were recoded as "successful" if their change score on the Musculoskeletal Health Questionnaire exceeded the minimal important change (explained below Table 2).

**Table 1. Baseline risk stratification groups:**

|  | Good function (6-10) | Poor function (0-5) |
| --- | --- | --- |
| Low pain (0-4) | **Low symptoms**  *(Risk group 1)* | **Poor Function**  *(Risk group 3)* |
| High pain (5-10) | **High Pain**  *(Risk group 2)* | **High Pain and Poor Function**  *(Risk group 4)* |

**Table 2. Scoring of outcomes at 3 months conditioned on baseline group:**

|  | Baseline Risk groups | | | |
| --- | --- | --- | --- | --- |
|  | 1 | 2 | 3 | 4 |
| 3 months | Low symptoms | High  Pain | Poor Function | High Pain and Poor Function |
| **Pain level at 3 months ≤3** | - | **1** | - | **1** |
| **Pain reduction from baseline ≥3** | - | **1** | - | **1** |
| **Function level at 3 months ≥8** | - | - | **1** | **1** |
| **Function improvement from base ≥3** | - | - | **1** | **1** |
| Sum |  |  |  |  |
| Scores added from only **one** of the cells below: | | | | |
| \| **Work Ability at 3 months (0-10)** \| \| \|  \|  \|  \| \| --- \| --- \| --- \| --- \| --- \| --- \| \| Poor \| Moderate \| Good/Excellent \|  \|  \| **Global perceived effect (1-7)** \| \| 0-5 \| 6-7 \| 8-10 \|  \|  \| \| **1** \| **2** \| **3** \| 1 \| Very much better \| \| - \| **1** \| **2** \| 2 \| Much better \| \| - \| - \| **1** \| 3 \| Minimal better \| \| - \| - \| - \| 4-7 \|  \| \|  \|  \|  \|  \|  \| | | | | |
| Total Sum |  | | | |
|  | | | | |
|  | Low symptoms | High  Pain | Poor  Function | High Pain and  Poor Function |
| **Total possible score** | 0-3 | 0-5 | 0-5 | 0-7 |
| **Scores defining successful outcome*** | **≥ 2** | **≥ 3** | **≥ 3** | **≥ 4** |

* Patients who did not reach the threshold for successful outcome, as defined in the last line of the table above but achieved a change score ≥6 (baseline to 3 months) on the Musculoskeletal Health Questionnaire and scored either 1 (very much better) or 2 (much better) on the Global Perceived Effect scale, were added as cases with "successful outcome".

**References**

1. Linton SJ, Nicholas M, MacDonald S. Development of a Short Form of the Örebro Musculoskeletal Pain Screening Questionnaire. Spine (Phila Pa 1976). 2011;36(22). https://doi.org/10.1097/brs.0b013e3181f8f775

2. Stratford P, Gill C, Westaway M, al. e. Assessing disability and change on individual patients: a report of a patient specific measure. Physiotherapy Canada. 1995;47(4):258-63. https://doi.org/10.3138/ptc.47.4.258

3. El Fassi M, Bocquet V, Majery N, Lair ML, Couffignal S, Mairiaux P. Work ability assessment in a worker population: comparison and determinants of Work Ability Index and Work Ability score. BMC Public Health. 2013;13(1):305. http://doi.org/10.1186/1471-2458-13-305

4. Dworkin RH, Turk DC, Farrar JT, Haythornthwaite JA, Jensen MP, Katz NP, et al. Core outcome measures for chronic pain clinical trials: IMMPACT recommendations. Pain. 2005;113(1-2):9-19. http://doi.org/10.1016/j.pain.2004.09.012

5. Hill JC, Kang S, Benedetto E, Myers H, Blackburn S, Smith S, et al. Development and initial cohort validation of the Arthritis Research UK Musculoskeletal Health Questionnaire (MSK-HQ) for use across musculoskeletal care pathways. BMJ Open. 2016;6(8):e012331. http://doi.org/10.1136/bmjopen-2016-012331
